# A Sensitive and Selective Electrochemical Sensor Based on an Iron-Based Nanocomposite-Modified Electrode for the Detection of Dopamine in Pork

**DOI:** 10.3390/foods14183145

**Published:** 2025-09-09

**Authors:** Jing Li, Luyao Wang, Jijie Shi, Xuelian Wu, Jing Zhang, Yuecheng Xu, Xinhui Wang, Xiaoqin Li

**Affiliations:** 1 College of Food and Biological Engineering, Chengdu University, Chengdu 610106, China; lijing@cdu.edu.cn (J.L.);; 2 Key Laboratory of Meat Processing of Sichuan Province, Chengdu University, Chengdu 610106, China; 3 Sichuan Institute of Food Inspection, Chengdu 610731, China; 4 Key Laboratory of Fine Chemicals and Surfactants, Sichuan Provincial University, Sichuan University of Science & Engineering, Zigong 643000, China

**Keywords:** iron-based nanocomposites, electrochemical sensor, dopamine, pork

## Abstract

Here, a highly sensitive and selective electrochemical sensor for dopamine (DA) determination in pork was developed. This sensor was fabricated using iron-based nanocomposites (Fe@(C-S-N)) to modify a glassy carbon electrode (GCE) substrate. The Fe@(C-S-N) nanocomposites were synthesized through a facile low-temperature chemical precipitation approach by adopting ferrous sulfate and melamine as raw materials, with their structural and compositional properties thoroughly investigated using multiple analytical techniques, including X-ray diffraction (XRD), electron microscopy (EM), X-ray photoelectron spectroscopy (XPS), and energy-dispersive spectra (EDS). Cyclic voltammetry (CV), chronocoulometry (CC), and differential pulse voltammetry (DPV) were adopted to investigate the electrochemical performance of the fabricated Fe@(C-S-N)/GCE sensor. Notably, the sensor demonstrated a linear response range from 0.05 to 100 μM with a minimal limit of detection (LOD) of 46 nM. Lastly, the fabrication sensor was used to determine DA in pork samples, showing an acceptable recovery of over 96.89%. Thus, the proposed sensor could offer an effective method for determining DA in pork.

## 1. Introduction

As the global leader in both meat production and consumption volumes [1], China is facing increasing public scrutiny regarding food safety standards. As a crucial catecholamine neurotransmitter, DA plays a vital role in neural networks, metabolism in mammals, and cardiovascular, renal, and hormonal system function [2]. Owing to its unique biological properties, which promote lean muscle development over adipose tissue accumulation in livestock, DA has been widely used as an unauthorized feed additive, commercially marketed as a “lean meat booster”, within the global animal husbandry sector for substantial financial gains [3]. Previous studies have shown that long-term consumption of livestock and poultry meat with DA residue is exceptionally harmful to the human body and has adverse side effects such as headaches, chest tightness, and nausea [4]. Therefore, the quantitative analysis of DA in meat samples has become significantly more important.

To date, DA and its analogs have been identified using thin-layer chromatography (TLC) [5], ion chromatography (IC) [6], capillary electrophoresis (CE) [7], enzyme-linked immunosorbent assay (ELISA) [8], HPLC [9], and GC-MS [10]. While these methods exhibit good selectivity and a low LOD, they are often time-consuming and require expensive instrumentation. Therefore, establishing a reliable method for detecting DA in meat samples is important. In contrast to the above techniques, electrochemical sensors have characteristics, such as a quick response, sensitive detection, ease of operation, lower detection costs, and no cumbersome sample pre-treatment, that are more suitable for the immediate detection of DA [11]. So far, two types of sensors have been explored: tyrosinase-based biosensors [12,13] and non-enzymatic DA sensors. Nevertheless, the disadvantages of enzyme inactivation, high costs, and a complex enzyme immobilization process limit the application of enzyme-based sensors [14]. In contrast, non-enzymatic DA sensors have drawn more attentions as DA can be detected by direct electrooxidation at the surface of nanomaterial-modified electrodes with low costs, high sensitivity, and long-term reliability [15]. Therefore, massive efforts have been undertaken to develop electrode-modified nanomaterials for non-enzymatic DA sensors.

Among the electrode-modified nanomaterials, metal-based nanomaterials have gained widespread applications for their excellent chemical stability, high conductivity, and remarkable electrocatalytic activity [16,17]. Owing to their advantages of low costs, multiple valence states, high electron transfer efficiency, good biocompatibility, and environmental friendliness, iron-based nanomaterials are among the most widely utilized modified materials in electrochemical sensors [18]. These materials comprise a broad range of compounds with distinct properties that can be used to improve the performance of electrochemical sensors, including iron oxides, iron nitride, iron sulfides, and other iron-based composites [19].

**Scheme 1:**
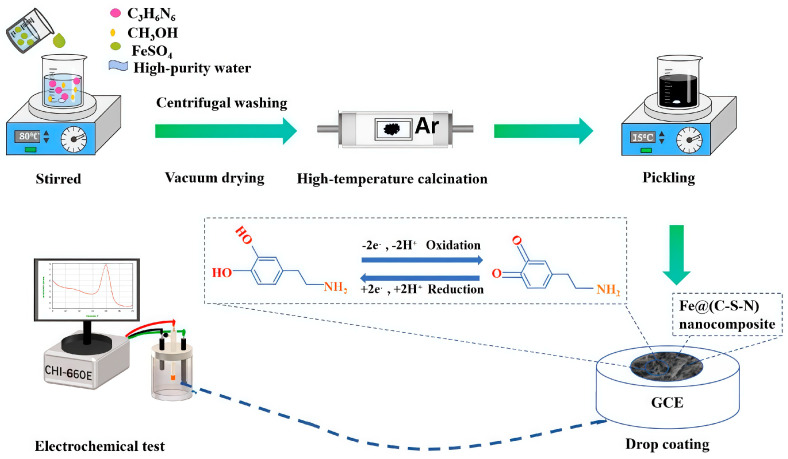
Outline of the synthesis of Fe@(C-S-N) nanocomposites for the DA electrochemical sensor.

In this work, a low-temperature chemical precipitation method with ferrous sulfate and melamine as raw materials was adopted to synthesize the Fe@(C-S-N) nanocomposites, including iron carbide, iron sulfide, iron nitride, and carbon nanotubes. And their electrochemical behaviors toward DA detection were explored (Scheme 1). The nanocomposites’ crystalline and morphological properties were characterized using XRD, EM, and other techniques. And the performance of the DA electrochemical sensor was optimized under various electrolyte pHs, accumulation times, and other experimental factors. Finally, DPV was employed as a detection methodology to determine DA with high selectivity, sensitivity, and stability. The practical applicability of this approach was validated by real sample analysis, yielding satisfactory recovery rates that confirmed its reliability for quantitative determination.

## 2. Materials and Methods

### 2.1. Chemicals and Instrument

Ferrous sulfate heptahydrate (FeSO_4_·7H_2_O) and melamine (C_3_H_6_N_6_) were purchased from the Adamasbeta Chemical Reagent Co., Ltd. (Shanghai, China). Dopamine hydrochloride (DA), ascorbate (AA), uric acid (UA), folic acid (FA), citric acid (CA), histidine (His), phenylalanine (Phe), Serine (Ser), L-Cysteine (L-Cys), tyrosine (Tyr), norepinephrine (NE), and isopropanol were bought from the Shanghai Aladdin Reagent Co., Ltd. (Shanghai, China). Methanol, zinc chloride (ZnCl_2_), niacin (NA), Catechol (C_6_H_6_O_2_), Bisphenol A (BPA), magnesium chloride (MgCl_2_), potassium nitrate (KNO_3_), anhydrous calcium chloride (CaCl_2_) and sodium chloride (NaCl) were produced by the Chengdu Kelong Chemical Co., Ltd. (Chengdu, China). High-purity water was used throughout the experiment. The crystallographic properties were analyzed using a DX-2700B powder X-ray diffractometer (Haoyuan, Dandong, China). Surface chemical composition analysis was conducted using a K-Alpha X-ray photoelectron spectrometer (Thermo Scientific, Waltham, MA, USA). The Zeiss Gemini 300 apparatus (Carl Zeiss, Thunstrasse, Germany) was used to acquire pictures from field emission scanning electron microscopy (FESEM). Transmission electron microscopy (TEM) images were captured from the JEOL-2100F transmission electron microscope (Japan Electronics Corporation, Tokyo, Japan).

### 2.2. Synthesis of the Fe@(C-S-N) Nanocomposites

Initially, 3.0 g of melamine was added slowly to 120 mL of a methanol-water mixture (V_methanol_/V_water_ = 1:2) at 80 °C with thorough stirring. Then, 40 mL of freshly made 0.405 M FeSO_4_ solution was gradually added dropwise to the aforementioned melamine solution while being stirred until it had completely dissolved. After that, the solution was sealed with plastic wrap and stirred at 80 °C for 2 h. After centrifugal washing, vacuum drying, and grinding, the as-obtained black powder was calcinated at 900 °C for 2 h with a heating rate of 5 °C min^−1^ under the protection of Ar atmosphere. Finally, the product labeled as Fe@(C-S-N) was obtained after treatment with a 0.5 M sulfuric acid solution for 6 h, washed with high-purity water, and dried in a 60 °C oven for 12 h.

### 2.3. Preparation of Fe@(C-S-N)/GCE and Electrochemical Test

First, 9.0 mg of Fe-@(C-S-N) was added to 0.5 mL of a isopropanol–water mixed solution (V_isopropanol_/V_water_ = 1:4) with the addition of 25 μL of Nafion (5 wt%), and then the mixture was ultrasonically treated to ensure its uniform dispersion. Meanwhile, the bare GCE (Ø = 3 mm) was thoroughly polished with alumina polishing powder and cleaned ultrasonically using high-purity water. Then, 3 μL of the above Fe@(C-S-N) suspension was dripped onto a clean GCE’s surface and dried overnight at room temperature to fabricate the Fe@(C-S-N)/GCE. Electrochemical experiments, such as cyclic voltammetry (CV), chronocoulometry (CC), and differential pulse voltammetry (DPV) were performed on the CHI660e electrochemical workstation (Shanghai CH Instruments Inc., Shanghai, China). Electrochemical impedance measurements were performed using an M204 electrochemical workstation (Autolab, Metrohm China Ltd., Beijing, China) over a frequency range of 0.1 Hz–100 kHz with an applied AC potential of 25 mV to evaluate the interfacial charge transfer characteristics. Every electrochemical test was conducted using a standard three-electrode setup at room temperature, comprising either an Fe@(C-S-N)-modified or unmodified GCE working electrode, paired with a platinum counter electrode (Ø = 3 mm) and an Ag/AgCl reference electrode.

### 2.4. Detection of DA in Meat Sample

The meat sample was prepared using the protein precipitation method with ZnCl_2_ [20]. Briefly, 5.0 g of fresh pork was mechanically homogenized with 10 mL of PBS solution (0.2 M, pH 7.0) to form a uniform slurry. The homogenate was subsequently treated with 10 mL of 1.0 M ZnCl_2_ solution and subjected to continuous agitation (100 rpm) for 10 min at ambient temperature. Following pH neutralization, the mixture was centrifuged (5000 rpm, 15 min, 4 °C), and the supernatant was filtered through a 0.22 μm membrane filter (Millex syringe filter). Before DA determination, 100 μL of supernatant was added to 900 μL of PBS electrolyte.

## 3. Results and Discussion

### 3.1. Characterization of the Synthesized Fe@(C-S-N) Nanocomposites

**Figure 1:**
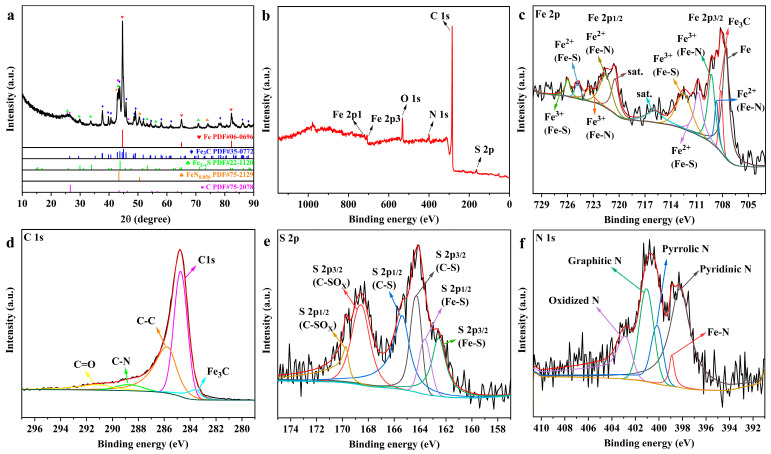
(**a**) XRD pattern of Fe@(C-S-N) nanocomposites; (**b**) large-scale and high-resolution XPS spectra of Fe@(C-S-N) nanocomposites for (**c**) Fe 2p, (**d**) C 1s, (**e**) S 2p, and (**f**) N 1s peaks.

Firstly, XRD was performed to clarify the composition of the prepared nanocomposite, and the result is shown in Figure 1a. The XRD pattern revealed sharp diffraction peaks at 2θ values of 44.6°, 64.9°, and 82.3°, corresponding to the (110), (200), and (211) crystallographic planes of α-Fe (JCPDS No. 06-0696), respectively [21]. The observed diffraction patterns at 2θ = 30.0°, 34.0°, 43.9°, 53.3°, and 73.3° were successfully assigned to the hexagonal pyrrhotite phase Fe_1−x_S (JCPDS No. 22-1120) [22]. The diffraction peaks at 43.4°, 50.6°, and 74.3° aligned with the (111), (200), and (220) crystal faces of FeN_0.056_ (JCPDS card No.75-2129) [23]. The other peaks corresponded to Fe_3_C (JCPDS card No. 35-0772) [24] and graphite carbon (JCPDS card No. 75-2078) [25], respectively.

The element composition and valence state of the Fe@(C-S-N) nanocomposites were inspected using XPS analysis. We can see that the nanocomposites mainly comprise Fe, C, S, N, and O elements in the XPS survey spectrum displayed in Figure 1b. Deconvolution analysis of the O 1s spectrum revealed two distinct components at binding energies of 531.1 eV and 533.7 eV, corresponding to C-O bonding configurations and hydroxyl species from surface-adsorbed water, respectively (Figure S1) [26]. The high-resolution Fe 2p XPS spectrum in Figure 1c shows ten distinct peaks. Through Gaussian–Lorentzian curve fitting, the 2p orbital of Fe can be divided into Fe^2+^ (Fe-S) peaks at 2p_1/2_ 724.7 eV and 2p_3/2_ 710.8 eV, Fe^3+^ (Fe-S) peaks at 2p_1/2_ 726.0 eV and 2p_3/2_ 712.8 eV [27], Fe^2+^ (Fe-N) peaks at 2p_1/2_ 721.7 eV and 2p_3/2_ 708.7 eV, and Fe^3+^ (Fe-N) peaks at 2p_1/2_ 723.6 eV and 2p_3/2_ 709.3 eV [28], respectively. The peaks at 707.3 eV and 708.0 eV are attributed to metallic Fe and FeC_3_ species. The peaks at both 716.7 eV and 720.6 eV were attributed to the satellite signals [27,28]. As shown in Figure 1d, the characteristic peak observed at 283.6 eV confirms the presence of Fe_3_C. Spectral deconvolution reveals four additional components at 284.7 eV (sp^2^ hybridized carbon), 285.8 eV (C-C bonds), 288.8 eV (C-N groups), and 291.4 eV (carbonyl functionalities), providing comprehensive evidence of the material’s complex carbon chemistry [24,29]. The C-C bond confirmed the presence of graphitic carbon, which was consistent with the XRD result. The high-resolution XPS analysis of S 2p orbitals (Figure 1e) reveals three characteristic doublets through spectral deconvolution. The first pair, observed at 162.7 eV (S 2p_3/2_) and 163.7 eV (S 2p_1/2_), corresponds to sulfur species in Fe-S coordination environments. A second doublet appears at 164.3 eV and 165.4 eV, indicating C-S covalent bonding configurations. Surface oxidation products are evidenced by the third doublet at 168.6 eV and 169.7 eV, assigned to various C-SOx (x = 2–4) species [30]. N 1s spectrum analysis (Figure 1f) demonstrates five nitrogen chemical states [31]: oxidized N (402.8 eV), graphitic N (401.2 eV), pyrrolic N (400.2 eV), Fe-N coordination (398.9 eV), and pyridinic N (398.2 eV) [32,33].

**Figure 2:**
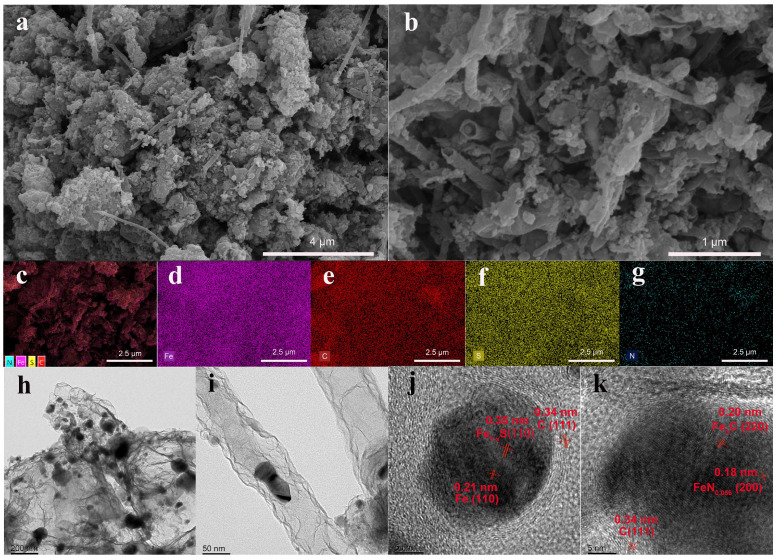
(**a**,**b**) FESEM images of Fe@(C-S-N) nanocomposites; (**c**–**g**) EDS mapping of Fe, C, S, and N elements in Fe@(C-S-N) nanocomposite; (**h**,**i**) TEM images; and (**j**,**k**) HRTEM images of Fe@(C-S-N) nanocomposites.

Scanning and transmission electron microscopy methods were used to examine the morphology and microstructural characteristics of the Fe@(C-S-N) nanocomposites. As seen from the FESEM images in Figure 2a,b, the nanocomposites mainly comprised nanotubes, nanoparticles, and nanoblocks. Nanotubes of different lengths were interwoven between the nanoparticles and the nanoblocks, which can not only provide a channel for electron transfer but also prevent the agglomeration of nanoparticles such as Fe_3_C [26]. Elemental mappings (panels c–g of Figure 2) indicate that Fe, C, S, and N elements are uniformly distributed within the Fe@(C-S-N) nanocomposites. The TEM images in Figure 2h,i also verify the mixed structural characteristics of the nanocomposite. Then, the HRTEM images (Figure 2j,k) further illustrate that the graphitic layers enclose the iron compound. The measured interplanar distance of approximately 0.34 nm between graphitic layers corresponds to the d-spacing of the (111) crystallographic plane in graphitic carbon structures. In Figure 2j, distinguishable lattice fringes with spacings of 0.21 nm and 0.35 nm are identified as the (110) planes of metallic Fe and pyrrhotite (Fe_1−x_S), respectively. Furthermore, Figure 2k demonstrates additional structural characteristics, with measured interplanar distances of 0.18 nm and 0.20 nm, corresponding to the (200) and (220) crystallographic planes of the FeN_0.056_ and Fe_3_C phases, respectively. These observations prove the structural correlation and interfacial interactions between these crystalline components.

### 3.2. Electrochemical Behavior

**Figure 3:**
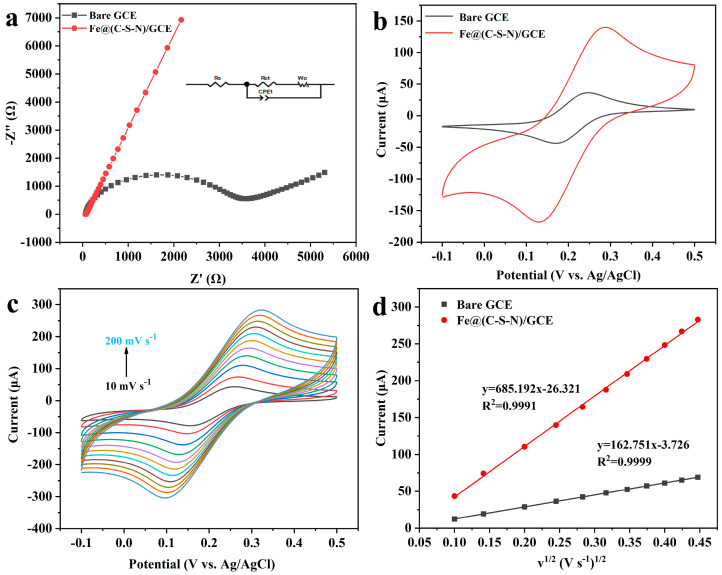
(**a**) EIS plot of bare GCE and Fe@(C-S-N)/GCE (inset: equivalent circuit image for EIS data); (**b**) CV diagrams of both bare GCE and Fe@(C-S-N)/GCE at 50 mV s^−1^; (**c**) CV diagrams of the Fe@(C-S-N)/GCE at different scan rates; and (**d**) linear fitting diagrams of Ip versus v^1/2^ of the Fe@(C-S-N)/GCE and the bare GCE.

To estimate the electrochemical performance of both the Fe@(C-S-N)/GCE and bare GCE, both the CV and EIS testing technologies were performed in 5 mM of [Fe (CN)_6_]^3−/4−^ solution with 0.1 M KCl. Figure 3a shows the Nyquist plots for the two electrodes. Rct, Rs, CPE1, and Wo are used to denote the charge transfer resistance, electrolyte solution resistance, constant phase angle element, and Warburg impedance, respectively. The value of Rct is quantitatively determined from the semicircular arc diameter in the Nyquist plot’s intermediate frequency domain, which depends on the insulating properties at the interface between the working electrode and the electrolyte. The Fe@(C-S-N)/GCE demonstrated a notably smaller Rct value of 53.22 Ω compared to the bare GCE’s 3500 Ω, suggesting that the synergistic interaction between elemental Fe, graphite carbon, and other iron-based compounds enhances electron transfer on the GCE surface. Furthermore, to evaluate the electrocatalytic activity of the Fe@(C-S-N)/GCE, the standard exchange current density (*I*_0_) can be estimated using Equation (2) [34]:

(1) $$I_{0}=\frac{RT}{nFR_{ct}}$$

where R denotes the gas constant (8.314 J mol^−1^·K^−1^), n stands for the number of electrons transferred for the [Fe (CN)_6_]^3-^/^4-^ system (n = 1), T is the absolute temperature (K), and F refers to the Faraday constant (96,485 C mol^−1^). The evaluated I_0_ of the Fe@(C-S-N)/GCE (482.74 µA cm^−2^) is significantly greater than that of the bare GCE (6.774 µA cm^−2^), indicating that Fe@(C-S-N) has much better intrinsic electrocatalytic activity. Hence, the Fe@(C-S-N)/GCE is suitable for developing electrochemical sensors. As displayed in Figure 3b, the redox peaks at the two electrodes facilitate the reversible reaction between [Fe(CN)_6_]^4−^ and [Fe(CN)_6_]^3−^. It is worth noting that the Fe@(C-S-N)/GCE exhibited higher redox peaks, profiting from the excellent electron transfer ability of the carbon nanotubes intercalated with the iron-based nanocomposites. Both (Figure 3c and Figure S2 reveal the CV diagrams for the two electrodes at different scan rates, respectively. From 10 to 200 mV s^−1^, the redox peak continuously increases as scan rates increase. Meanwhile, the anode and cathode potentials move in the opposite directions, revealing a quasi-reversible redox process occurring on the Fe@(C-S-N)/GCE. The effective areas of the two electrodes can be calculated according to the Randles-Sevcik equation as follows [26]:

I_p_ = 2.69 × 10^5^·*A*D^1/2^n^3/2^v^1/2^c_0_ (2)

Here, I_p_ signifies the anodic peak current (A), *A* represents the electroactive surface area of the electrode (cm^2^), D denotes the electrolyte diffusion coefficient (6.67 × 10^−6^ cm^2^ s^−1^), n represents the number of electrons transferred for the reaction involving [Fe (CN)_6_]^3−/4−^ (n = 1), v indicates the scan rate (V s^−1^), and c_0_ refers to the electrolyte concentration. By analyzing the CV data (Figure 3d), the active areas were calculated to be 0.047 cm^2^ for the bare GCE and 0.197 cm^2^ for the Fe@(C-S-N)/GCE. This apparent increase in the electroactive area demonstrates that Fe@(C-S-N) nanocomposites possess superior electroconductivity and can provide more electroactive sites for the modified GCE.

### 3.3. Optimized Experimental Conditions

**Figure 4:**
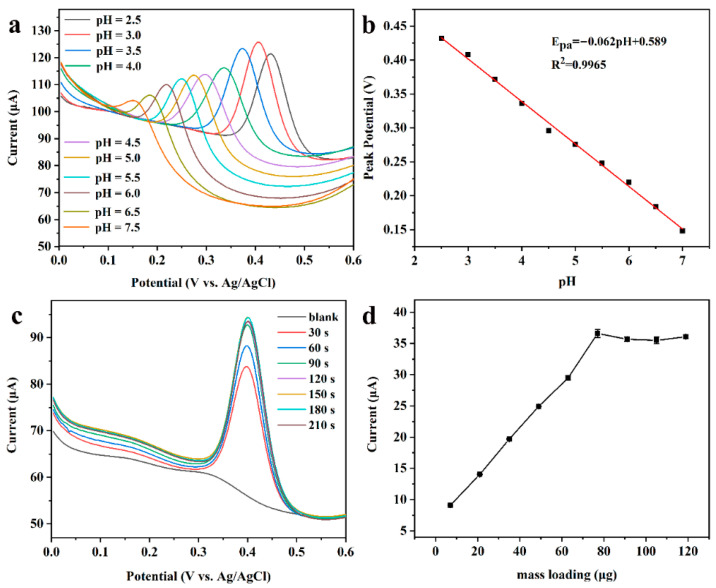
DPV diagrams of 30 μM DA at Fe@(C-S-N)/GCE with (**a**) different pHs and (**c**) various accumulation times; (**b**) linear fitting diagram between peak potential (E_pa_) and pH; (**d**) relationship between oxidation peak current and the Fe@(C-S-N) nanocomposites’ mass loading on the surface of the GCE (n = 3).

The effects of electrolyte pH, accumulation time, and the load mass of the active substance on the surface of the bare GCE were investigated using DPV. The PBS electrolyte’s pH range of 2.5 to 7.0 influences both the peak current and peak potential, as seen in Figure 4a. We can see that the ideal pH of 3.0 is where the largest oxidation peak current occurs. The peak potentials move in the negative direction as the pH increases, suggesting that the electrocatalytic oxidation toward DA at the Fe@(C-S-N)/GCE involves proton transfer during the electrode’s reaction process [35]. Figure 4b demonstrates a strong linear correlation between E_pa_ and pH, represented by E_pa_ = −0.062pH + 0.590 (R^2^ = 0.9965). The obtained slope of −0.062 V pH^−1^ demonstrates excellent agreement with the theoretically predicted Nernstian value of −0.059 V pH^−1^, confirming that the transfer of protons was accompanied by an equal transfer of electrons [36]. As accumulation time increased, the oxidation peak currents clearly increased (Figure 4c). Once the accumulation time exceeded 90 s, the oxidation peak currents remained almost constant. For subsequent experiments, 90 s was chosen as the accumulation time in consideration of both working efficiency and detection sensitivity. Furthermore, the mass of the modified material being loaded onto the surface of the bare GCE should be controlled in an optimal range. Excessive drip-coated amounts will cause agglomeration of the material, while too small a load cannot provide enough reactive sites. As seen from Figure 4d, the oxidation peak current reached its maximum value when the drip coating amount reached 80 μg, so 80 μg was chosen as the drip coating amount in the subsequent experiments.

### 3.4. Study of the Reaction Mechanism

**Figure 5:**
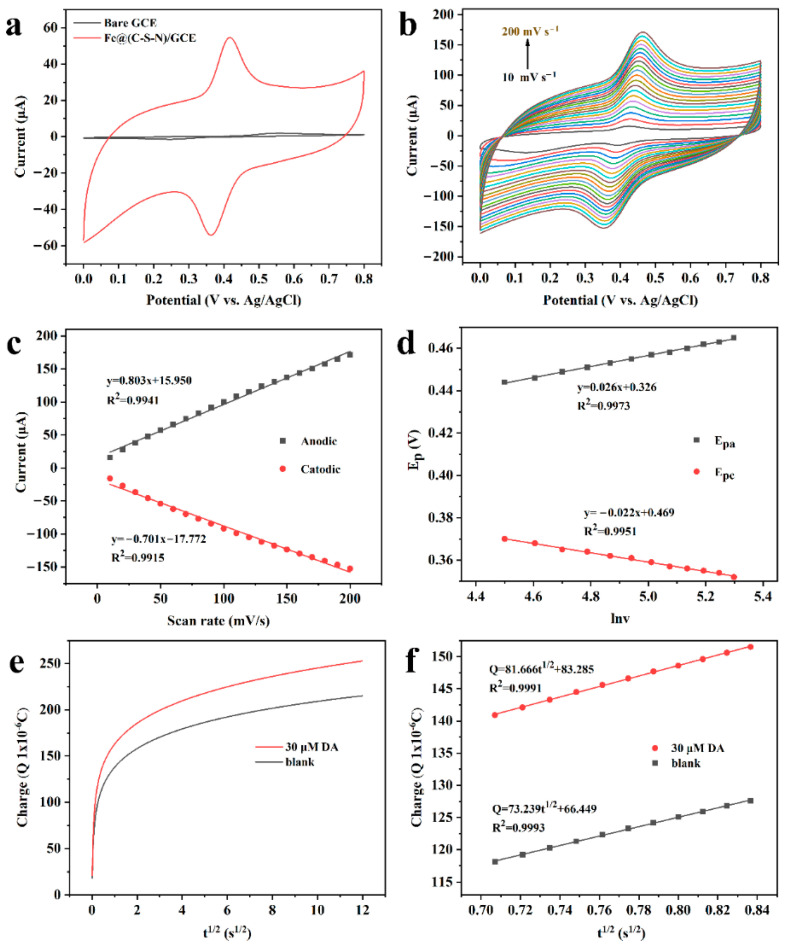
(**a**) CV diagram of the bare GCE and the Fe@(C-S-N)/GCE. (**b**) CV curves of the Fe@(C-S-N)/GCE at various scan rates. (**c**) The linear fitting diagram between v and I_p_. (**d**) The relationship between lnv and Ep; the electrolyte comprised 0.2 M PBS (pH = 3.0) containing 100 μM DA. (**e**) The Q-t diagram of the Fe@(C-S-N)/GCE in 0.2 M PBS (pH = 3.0) with or without 30 μM DA. (**f**) Linear relationship of Q versus t^1/2^.

The electrochemical responses of 100 μM DA on the Fe@(C-S-N)/GCE and the bare GCE electrodes were evaluated through CV at 50 mV s^−1^. The redox peak current on the modified electrode was significantly more robust than on the bare GCE, as demonstrated in Figure 5a. Meanwhile, the redox peak potential difference (ΔE_p_) for the Fe@(C-S-N)/GCE decreased to 54 mV, highlighting the improved sensitivity and enhanced reversibility of the Fe@(C-S-N) nanocomposites [37]. CV was also employed to elucidate the catalytic behavior and reaction mechanism of DA on the Fe@(C-S-N)/GCE. Figure 5b reveals that the oxidation and reduction peak potentials shift positively and negatively with increasing scan rates, respectively, while the peak currents intensify. In Figure 5c, we can see that the peak current (I_p_) has a strong linear relationship with the corresponding v: I_pa_ = 0.803v + 15.950 (R^2^ = 0.9941) and I_pc_ = −0.701v − 17.772 (R^2^ = 0.9915), revealing that the behavior of the DA molecules is primarily dominated by the surface adsorption-controlled process [38]. Furthermore, the linear relationship between peak potential (E_p_) and the natural logarithm of the scan rate (lnv) is illustrated in Figure 5d. The following fitted linear equations were derived: E_pa_ = 0.026lnv + 0.326 (R^2^ = 0.9973) and E_pc_ = −0.022lnv + 0.469 (R^2^ = 0.9951). The electron transfer kinetic parameters of DA on the Fe@(C-S-N)/GCE can be calculated according to the Laviron relation, as given in Equation (3), and the linear regression equations between lnv and E_p_, as shown in Equations (4) and (5).

(3) $$\log K_{s}=\alpha \log\left(1-\alpha \right)+\left(1-\alpha \right)\log\alpha -\log\left(\frac{RT}{nF\upsilon }\right)-\frac{\alpha \left(1-\alpha \right)nF∆E_{p}}{2.3RT}$$

(4) $$E_{pa}=E^{\theta }+\left(\frac{RT}{\alpha nF}\right)\ln\nu$$

(5) $$E_{pc}=E^{\theta }-\left[\frac{RT}{\left(1-\alpha \right)nF}\right]\ln\nu$$

In this context, K_s_ denotes the standard electron transfer rate constant, while α symbolizes the charge transfer coefficient. The parameter R corresponds to the universal gas constant, T indicates the absolute temperature, and F refers to the Faraday constant. Additionally, E^θ^ represents the standard peak potential, n stands for the number of electron transfers, and v represents the scan rate. Based on the calculations, the values of α and n were determined to be 0.457 and 1.967, respectively, indicating that the oxidation reaction of DA on the modified electrode involves two electron transfers. With v = 50 mV s^−1^ and ΔE_p_ = 0.054 V, the calculated value of K_s_ was 0.860 s^−1^. This value is higher than those reported in previous studies [39], highlighting the accelerated electron transfer kinetics facilitated by the Fe@(C-S-N)/GCE’s surface.

To further verify the adsorption characteristics and catalytic performance of the Fe@(C-S-N)/GCE toward DA, a chronocoulometric measurement was performed (Figure 5e). We can observe two nearly parallel fitting lines in Figure 5f, confirming that the reaction was controlled by adsorption. The regression equations are, specifically, Q (10^−6^ C) = 81.666 t^1/2^ (s^1/2^) + 83.285 (R^2^ = 0.9991) and Q (10^−6^ C) = 73.239 t^1/2^(s^1/2^) + 66.449 (R^2^ = 0.9993), based on the formula (Equation (6)) provided by the theory of Anson and Laviron [40]:

Q_ads_ = nFAΓ* (6)

Here, n represents the electron transfer number for DA (n = 2), A is the active surface area of the Fe@(C-S-N)/GCE (0.197 cm^2^), F refers to Faraday’s constant (96,485.34 C mol^−1^), and Q_ads_ signifies the Faradaic charge, which can be obtained from the difference between the intercepts of the two Q-t^1/2^ fitting lines on the y-axis. The adsorption capacity (Γ*) of DA on the Fe@(C-S-N)/GCE was calculated to be 4.4298 × 10^−10^ mol cm^−2^, enhancing the electrochemical response.

### 3.5. DPV Determination of DA

**Figure 6:**
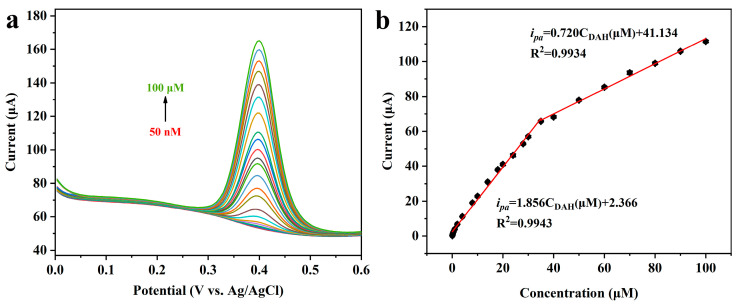
(**a**) DPV of the Fe@(C-S-N)/GCE in 0.2 M PBS (pH = 3) with different concentrations of DA; (**b**) the corresponding linear relationship between Ip and C_DA_.

DPV was used to evaluate the Fe@(C-S-N)/GCE sensor’s role in detecting DA. As illustrated in Figure 6a, the oxidation peak current significantly increased with rising DA concentrations. Figure 6b demonstrates that the current response of the Fe@(C-S-N)/GCE sensor correlates linearly with DA concentration in two different ranges (0.05 μM to 35 μM and 35 μM to 100 μM). The corresponding linear regression equations are expressed as I_p_ = 1.929C_DA_ + 0.821 (R^2^ = 0.9935) and I_p_ = 0.741C_DA_ + 39.854 (R^2^ = 0.9953), respectively. The LOD for DA detection was determined to be 0.046 μM using the formula LOD = 3σ/s, where σ is the standard deviation derived from 10 measurements of a blank solution (without DA) and s represents the slope of the calibration curve. The performance of the Fe@(C-S-N)/GCE sensor was benchmarked against other electrochemical sensors, as detailed in Table 1. The results highlight its advantages, including its broad linear detection range and low LOD, underscoring its potential for sensitive DA detection.

### 3.6. Effect of Interferences, Repeatability, Reproducibility, and Stability

**Figure 7:**
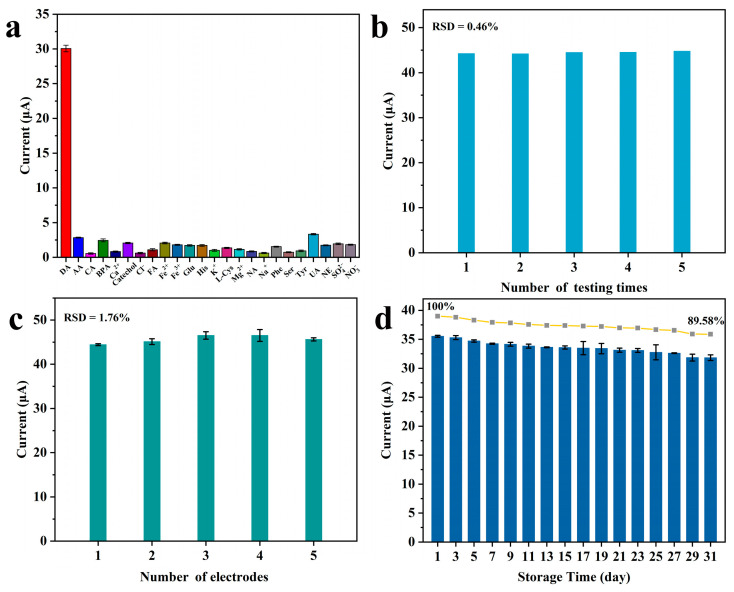
The (**a**) selectivity, (**b**) repeatability, (**c**) reproducibility, and (**d**) stability of the Fe@(C-S-N)/GCE toward DA in 0.2 M PBS (pH = 3.0).

The anti-interference and selectivity of the Fe@(C-S-N)/GCE sensor were investigated by detecting 20 μM DA in the presence of a 10-fold excess of various ions and compounds, including Na^+^, K^+^, Ca^2+^, Mg^2+^, Fe^2+^, Fe^3+^, Cl^−^, NO_3_^−^, SO_4_^2−^, L-Cys, Phe, Tyr, His, Ser, FA, NA, CA, Glu, BPA, Catechol, UA, NE, and AA. As illustrated in Figure 7a, the current response generated by DA is considerably higher than that of the interfering substances, which indicates that the Fe@(C-S-N)/GCE sensor possesses good selectivity and anti-interference capabilities. In Figure 7b, one Fe@(C-S-N)/GCE was repeated to test its repeatability. The RSD was calculated to be 0.46%. Additionally, the reproducibility of the Fe@(C-S-N)/GCE sensor was also examined via DPV with five Fe@(C-S-N)/GCEs in the same electrolyte (Figure 7c), and the RSD was 1.76%. The obtained results revealed their excellent repeatability and reproducibility. The long-term stability of the fabricated Fe@(C-S-N)/GCE sensor was evaluated over 31 days, and the result is displayed in Figure 7d. On the 31st day, the current remained at 89.58% of its initial value, indicating the excellent stability of the Fe@(C-S-N)/GCE.

### 3.7. Real-Time Analysis of DA

**Figure 8:**
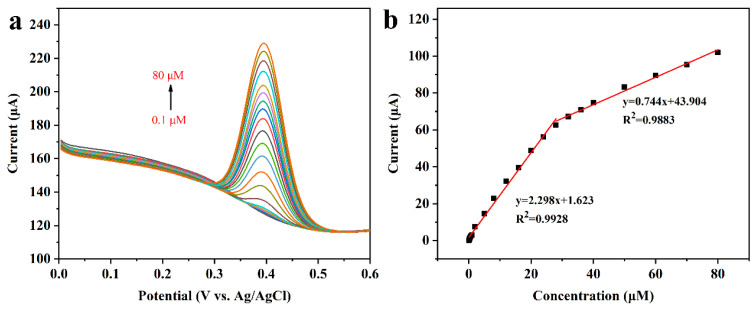
(**a**) DPV response of spiked DA in pork treatment liquid; (**b**) linear concentration fitting plot.

The standard addition method was adopted to study the feasibility and reliability of the Fe@(C-S-N)/GCE sensor for detecting DA in actual pork samples. Figure 8a shows the DPV response relationship of DA in actual pork samples, and the fitting curve is displayed in Figure 8b. As indicated in Table 2, the recovery rate for DA ranged from 96.89% to 105.19%, and the RSD values were all below 2.90%. These results suggest that the prepared Fe@(C-S-N)/GCE sensor is promising for detecting DA in pork.

## 4. Conclusions

In this work, a novel electrochemical sensor was fabricated by modifying the GCE with Fe@(C-S-N) nanocomposites, which were synthesized through a simple and efficient low-temperature chemical precipitation approach. The obtained Fe@(C-S-N)/GCEs exhibited high electrocatalytic activity for DA detection. Under optimized experimental conditions, the Fe@(C-S-N)/GCE showed good electrochemical performance regarding a broad linear detection range, a low LOD, high selectivity, repeatability, reproducibility, and long-term stability in DA determination. Furthermore, the practical applicability of the sensor was confirmed through the successful determination of DA levels in real meat samples, achieving highly satisfactory results.

## Figures and Tables

**Table 1:** DA detection performance comparison of various electrochemical sensors.

Electrode	Detection Method	Linear Range (μM)	LOD (μM)	Ref.
Fe_3_C@NGCSs/GCE	DPV	1.2–120.8	0.34	[41]
Fe/Fe_3_N-900/GCE	DPV	0.09–270; 297–455.5	0.97	[26]
COF-1/GCE	DPV	1–500	0.5	[42]
LCO-G/GCE	DPV	0.020–20	0.004	[39]
PSS/Chitosan/Graphene/SPE	DPV	0.05–70	0.29	[43]
Fe/Fe_3_C@CNTs/GCE	DPV	0.05–40	0.01	[44]
SPCE-Fe_3_O_4_/SPEEK	SWV	5–50	7.1	[45]
CuO-MgO	I-t	10–100	6.4	[46]
pGO-GNP-ITO	CV	0.1–30	1.28	[47]
PtNi@MoS_2_/GCE	DPV	0.5–150	0.1	[48]
Fe@(C-S-N)/GCE	DPV	0.05–35; 35–100	0.046	This work

NGCSs: N-doped graphitic carbon sheets; COF-1: covalent organic framework; LCO-G: lithium cobalt oxide mixed with graphite powder; PSS: polystyrene sulfonate; SPE: screen-printed electrode; CNTs: carbon nanotubes; SPCE: screen-printed carbon electrodes; SPEEK: sulfonated polyether ether ketone; GNPs: gold nanoparticles; ITO: indium tin oxide.

**Table 2:** DA content determination in the pork treatment liquid sample.

Sample	Labeled (μM)	Found (μM)	Recovery (%)	RSD (%)
Sample 1	3	2.91 ± 0.08	96.89 ± 2.75	2.84
Sample 2	7	7.36 ± 0.09	105.19 ± 1.35	1.67
Sample 3	17	16.6 ± 0.31	97.67 ± 1.88	0.91

## Data Availability

The original contributions presented in the study are included in the article/Supplementary Material, further inquiries can be directed to the corresponding authors.

## Supplementary Materials

The following supporting information can be downloaded at https://www.mdpi.com/article/10.3390/foods14183145/s1: Figure S1: The high-resolution XPS spectrum of the O 1s peak; Figure S2: CV diagrams of the bare GCE at scan rates from 10 to 200 mV s^−1^ in 5 mM [Fe (CN)_6_]^3-/4-^ solution containing 0.1 M KCl; Figure S3: DPV diagrams of the Fe@(C-S-N)/GCE’s response to 20 μM of DA and 200 μM of interferents; Figure S4: (a) The DPV curves obtained using different potential increments, (b) The relationship between oxidation peak current and potential increment; Figure S5: (a) The DPV curves obtained using different pulse amplitudes, (b) The relationship between oxidation peak current and pulse amplitude; Figure S6: (a) The DPV curves obtained using different pulse widths, (b) The relationship between oxidation peak current and pulse width.
