# Supplementary material for: A Sensitive and Selective Electrochemical Sensor Based on an Iron-Based Nanocomposite-Modified Electrode for the Detection of Dopamine in Pork
Source: Foods. 2025 Sep 9;14(18):3145. doi: 10.3390/foods14183145 (PMC12468867; doi:10.3390/foods14183145)
Supplement: Supplementary file 1 [file foods-14-03145-s001.zip › foods-3788942-supplementary.pdf]

Supplementary Materials

A sensitive and selective electrochemical sensor based on iron-based nanocomposite modified electrode for the detection of dopamine in pork

Jing Li[1, 2], Luyao Wang[1], Jijie Shi[1, 3], Xuelian Wu[1], Jing Zhang[1, 4],Yuecheng Xu[3], Xinhui Wang[1, 2*], Xiaoqin Li[1*]

1 College of Food and Biological Engineering, Chengdu University, Chengdu, 610106, China
2 Key Laboratory of Meat Processing of Sichuan Province, Chengdu 3 Sichuan Institute of Food Inspection, Chengdu 610731, China
4 Key Laboratory of Fine Chemicals and Surfactants in Sichuan Provincial University, Sichuan University of Science & Engineering, Zigong, 643000, China
* Correspondence: wangxinhui19820319@163.com (X. W.), lixiaoqin0321@163.com (X. L.)

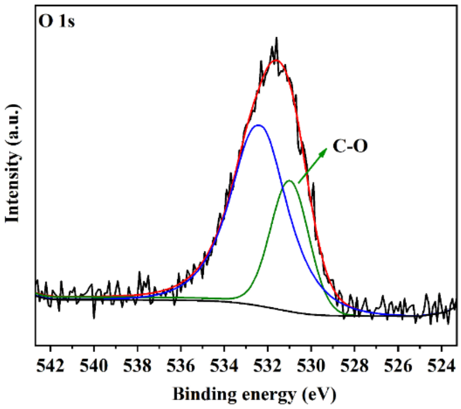

Figure S1 The high-resolution XPS spectrum of O 1s peak.

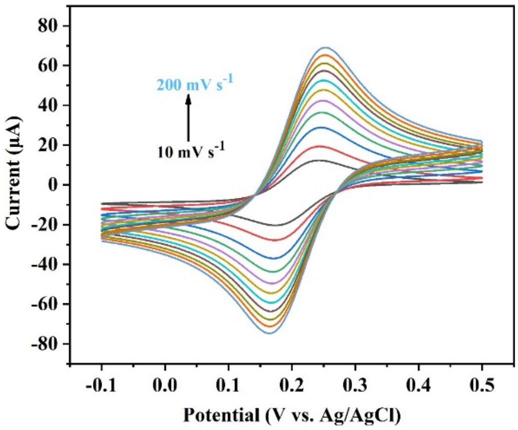

Figure S2 CV diagrams of bare GCE at scan rate from 10 to 200 mV s^{-1} in 5 mM $[Fe(CN)_6]^{3-/4-}$ solution containing 0.1 M KCl.

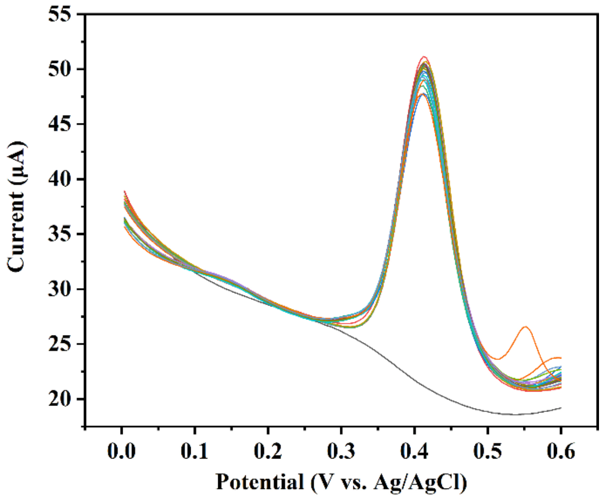

Figure S3 DPV diagrams of the Fe@(C-S-N)/GCE to 20 μM DA and 200 μM interferents.

We have evaluated the optimal working parameters for DPV measurements. Optimization of this method was performed via changing three variables: the potential increment Incr E(V), the pulse width (sec), or the pulse amplitude (V).

Figure S4a shows the impact of the optimization of potential increment (Incr E) on the detection performance of DA. From Figure S4b, we could find that the oxidation peak current is negatively correlated with the Incr E. The increase in current within the range of 0.015 - 0.004 V is relatively gentle, and becomes more obvious when it reaches 0.002 V. However, the test time spent on a single test at 0.001 V is more than twice that at 0.002 V, and the current does not show a significant increase. Therefore, considering both the test performance and the test time, the Incr E of 0.002 V was ultimately selected.

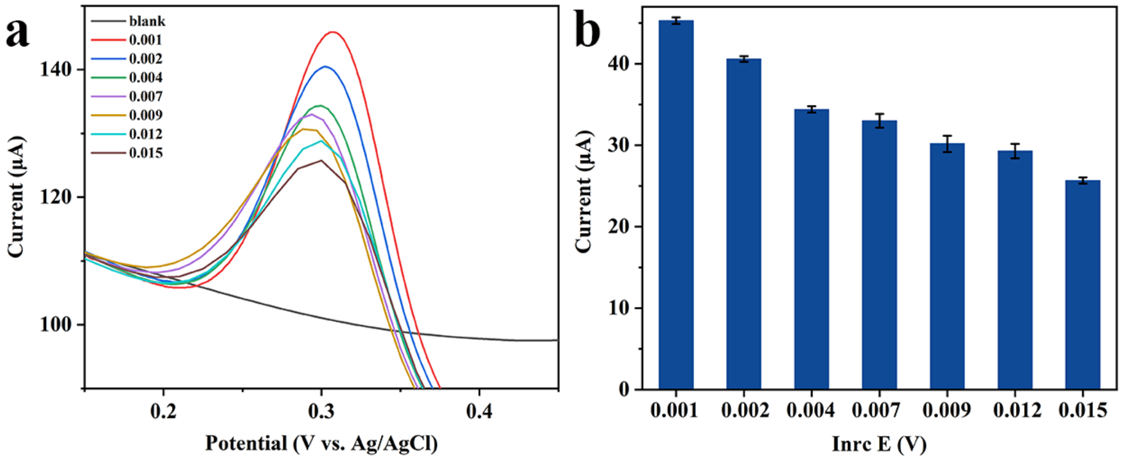

Figure S4 (a) The DPV curves obtained using different potential increments, (b) The relationship between oxidation peak current and potential increment

The relationship between pulse amplitude and response current is presented in Figure S5. Although the oxidation peak current significantly increased with the increase in pulse amplitude, the oxidation peak potential of DA showed a significant negative shift under this condition, which affected the subsequent analysis of the reaction mechanism.

Therefore, the original parameters (0.05 V) of DPV were selected.

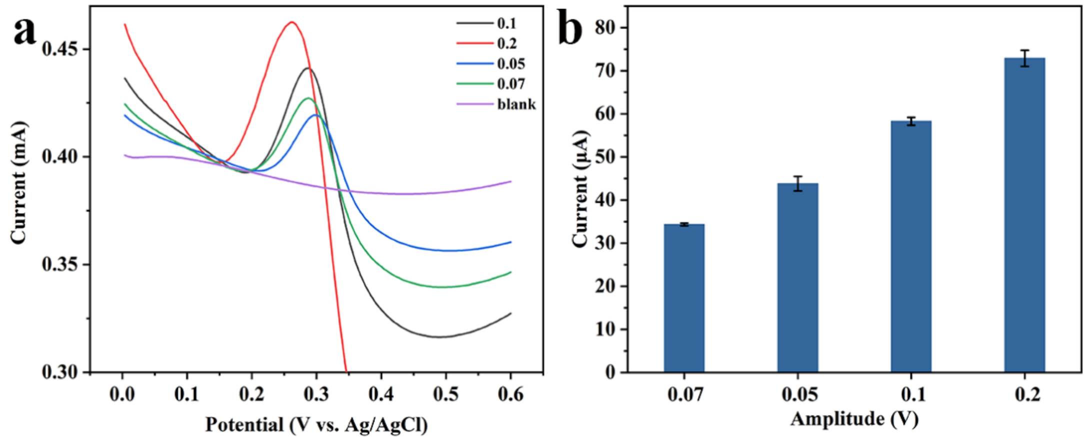

Figure S5 (a) The DPV curves obtained using different pulse amplitudes, (b) The relationship between oxidation peak current and pulse amplitude.

Figure S6 illustrates the relationship between pulse width and oxidation peak current. The oxidation peak current shows a trend of increasing first and then decreasing with the change of pulse width parameters. It reaches its maximum when the pulse width is 0.01 s, Hence, a pulse width of 0.01 s was chosen in the subsequent experiments.

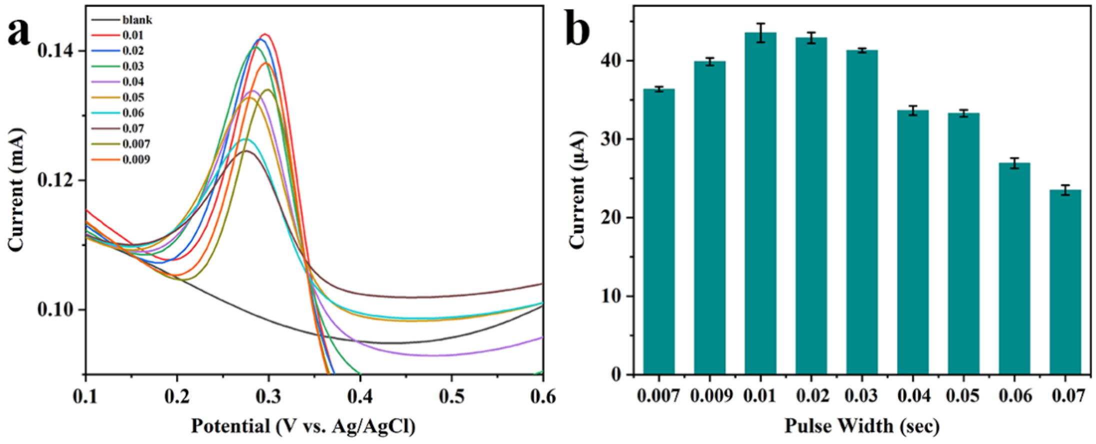

Figure S6 (a) The DPV curves obtained using different pulse widths, (b) The relationship between oxidation peak current and pulse width.
